# B7-H3 specific CAR-T cells exhibit potent activity against prostate cancer

**DOI:** 10.1038/s41420-023-01453-7

**Published:** 2023-05-06

**Authors:** Shibao Li, Miaomiao Zhang, Meng Wang, Haiting Wang, Han Wu, Lijun Mao, Meng Zhang, Huizhong Li, Junnian Zheng, Ping Ma, Gang Wang

**Affiliations:** 1 Department of Laboratory Medicine, Affiliated Hospital of Xuzhou Medical University, Xuzhou, Jiangsu China; 2 Department of Medical Technology, Xuzhou Medical University, Xuzhou, Jiangsu China; 3 Cancer Institute, Xuzhou Medical University, Xuzhou, Jiangsu China; 4 Center of Clinical Oncology, Affiliated Hospital of Xuzhou Medical University, Xuzhou, Jiangsu China; 5 Jiangsu Center for the Collaboration and Innovation of Cancer Biotherapy, Cancer Institute, Xuzhou Medical University, Xuzhou, Jiangsu China; 6 Department of Urology, Affiliated Hospital of Xuzhou Medical University, Xuzhou, Jiangsu China

**Keywords:** Targeted therapies, Preclinical research

## Abstract

B7-H3 is an attractive target for immunotherapy because of its high expression across multiple solid tumors, including prostate cancer, and restricted expression in normal tissues. Among various types of tumor immunotherapy, chimeric antigen receptor T (CAR-T) cell therapy has shown remarkable success in hematological tumors. However, the potency of CAR-T cell therapy in solid tumors is still limited. Here, we examined the expression of B7-H3 in prostate cancer tissues and cells and developed a second-generation CAR that specifically targets B7-H3 and CD28 as costimulatory receptor to explore its tumoricidal potential against prostate cancer in vitro and in vivo. The high expression of B7-H3 was detected on both the surface of PC3, DU145 and LNCaP cells and prostate cancer tissues. B7-H3 CAR-T cells efficiently controlled the growth of prostate cancer in an antigen-dependent manner in vitro and in vivo. Moreover, tumor cells could induce the proliferation of CAR-T cells and the release of high levels of cytokines of IFN-γ and TNF-α in vitro. Results demonstrated that B7-H3 is a potential target for prostate cancer therapy that supports the clinical development of B7-H3 specific CAR-T cells for prostate cancer.

## Introduction

The incidence of prostate cancer (PCa) continues to increase annually, and PCa has become the second leading malignancy in males [1] and the fifth most common tumor worldwide [2]. Radical surgery and castration are major treatment strategies for early-stage prostate cancer. However, the recurrence rate of PCa is high, and a certain number of patients will progress to the advanced castration-resistant PCa (CRPC) stage and eventually develop into metastatic castration-resistant PCa (mCRPC) [3]. Endocrine therapy is considered palliative because it can alleviate symptoms but fail to cure the disease. Chemotherapy alone presents limited efficacy and is hindered by drug resistance, while radiotherapy often shrinks the PCa tumor with evident complications. Following the FDA approval of sipuleucel-T for prostate treatment, some studies have evaluated the role of immunotherapy drugs in prostate cancer [4, 5] but found no significant effect [6–8]. Novel therapeutic strategies are urgently necessary to provide durable disease control and improve the survival of patients with PCa.

Cancer immunotherapy has become an effective regimen alone or in combination with other treatments, such as surgery, radiotherapy, chemotherapy, and targeted therapy. It activates host immune cells, especially T cells, to target tumor cells specifically. Among the cancer immunotherapies, chimeric antigen receptor (CAR) T cell therapy has overcome cancer immune tolerance and progressed considerably in liquid cancer treatment. CAR is an artificial T cell receptor consisting of tumor antigen-binding domain (such as single-chain fragment variable [scFv]) fused with T cell costimulatory and activating motifs [9]. Hence, CAR-modified T cell (CAR-T) could specifically recognize and kill tumor cells without an antigen-presenting process and major histocompatibility complex (MHC) restriction. CAR-T cell therapy was initially used in hematological malignancies and has shown high remission rates and persistence in chronic lymphocytic leukemia (CLL), acute lymphocytic leukemia (ALL), and refractory B-cell lymphoma [10–15]. It also demonstrated promising results in early clinical trials for other hematological malignancies, including multiple myeloma [16]. Clinical trials of CAR-T cell therapy for solid tumors, such as breast, liver, ovarian, and lung cancer, are increasing [17]. The target antigen of CAR-T cell therapy should be overexpressed on the surface of cancer cells in most patients and demonstrate no or low expression in normal tissues; thus, CAR-T cells can exert cancer-specific immune response without damaging normal tissues and organs [18, 19].

The type I transmembrane protein B7-H3, also called CD276, is highly expressed in various solid tumors, such as prostate cancer, renal cell carcinoma, non-small cell lung cancer, and breast cancer [20], but expressed at low levels in normal human tissues. It contributes to tumor immune evasion [21] and metastasis [22] and is associated with disease progression [23], recurrence, poor prognosis [24], and drug resistance. Roth et al. performed immunohistochemical analysis of tissue samples from PCa patients and revealed that B7-H3 is expressed in all tissues and its expression is associated with tumor aggressiveness, metastasis, and disease recurrence. Zang et al. showed that high expression of B7-H3 in PCa tissue is associated with tumor metastasis, postoperative recurrence, and high mortality. Hence, B7-H3 is a potential therapeutic target for prostate cancer. To date, multiple clinical trials have shown that monoclonal, bispecific, and drug-conjugated B7-H3 antibodies (MGA271 and 8H9) are safe and effective for the treatment of advanced malignant tumors [25–29]. A number of therapies targeting B7-H3 are already in preclinical or clinical trial stages [30]. B7-H3 CAR-T cells have been effective in solid tumors, such as neuroblastoma, ovarian cancer, pancreatic ductal adenocarcinoma, and melanoma [31, 32]. Furthermore, B7-H3 CAR-T cells significantly repress tumor growth in syngeneic tumor models and multiple preclinical studies without apparent toxicity [33]. Therefore, CAR-T targeting B7-H3 might be a promising therapeutic strategy for prostate cancer.

We first constructed a second-generation CAR targeting B7-H3 with humanized scFv from 8H9 and verified that B7-H3 CAR-T cells kill B7-H3-positive PCa cells in an antigen-dependent manner in vitro. In addition, infusion of B7-H3 CAR-T cells also significantly inhibited the growth of DU145 xenograft tumors in NCG mice. This finding suggested that B7-H3 targeted CAR-T therapy might be a novel immunotherapeutic strategy for PCa.

## Results

### Overexpression of B7-H3 in prostate cancer cell lines and tumor tissues

**Fig. 1:**
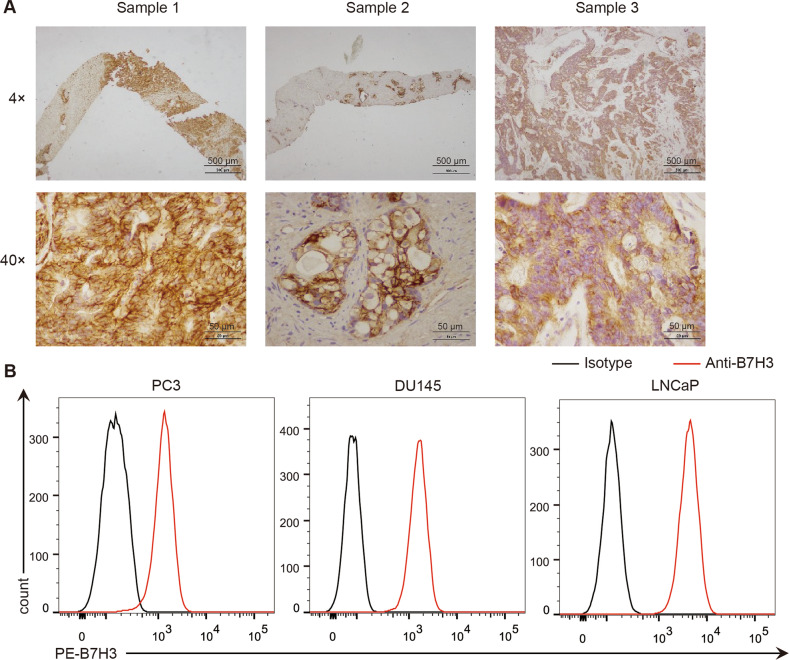
Expression of B7-H3 protein in prostate cancer tissues and cells. **A** Expression of B7-H3 protein in tissue samples of prostate cancer patients was detected through immunohistochemical analysis (Scale bar, 4 × 500 μm, 40 × 50 μm). **B** Flow cytometry was used to detect the expression of B7-H3 on the surface of prostate cancer cells.

We examined the expression of the B7-H3 molecule in PCa tissues and cell lines to assess whether it could be a target for PCa. Immunohistochemistry of tumor tissue from three clinical prostate cancer patients revealed the high expression of B7-H3 (Fig. 1A). The result of flow cytometry (FCM) showed that B7-H3 is highly expressed on the surface of prostate cancer cell lines PC3, DU145, and LNCaP (Fig. 1B). These results indicated that B7-H3 is a potential PCa target for the development of novel therapeutic strategies.

### Construction of CAR-T cells targeting B7-H3

**Fig. 2:**
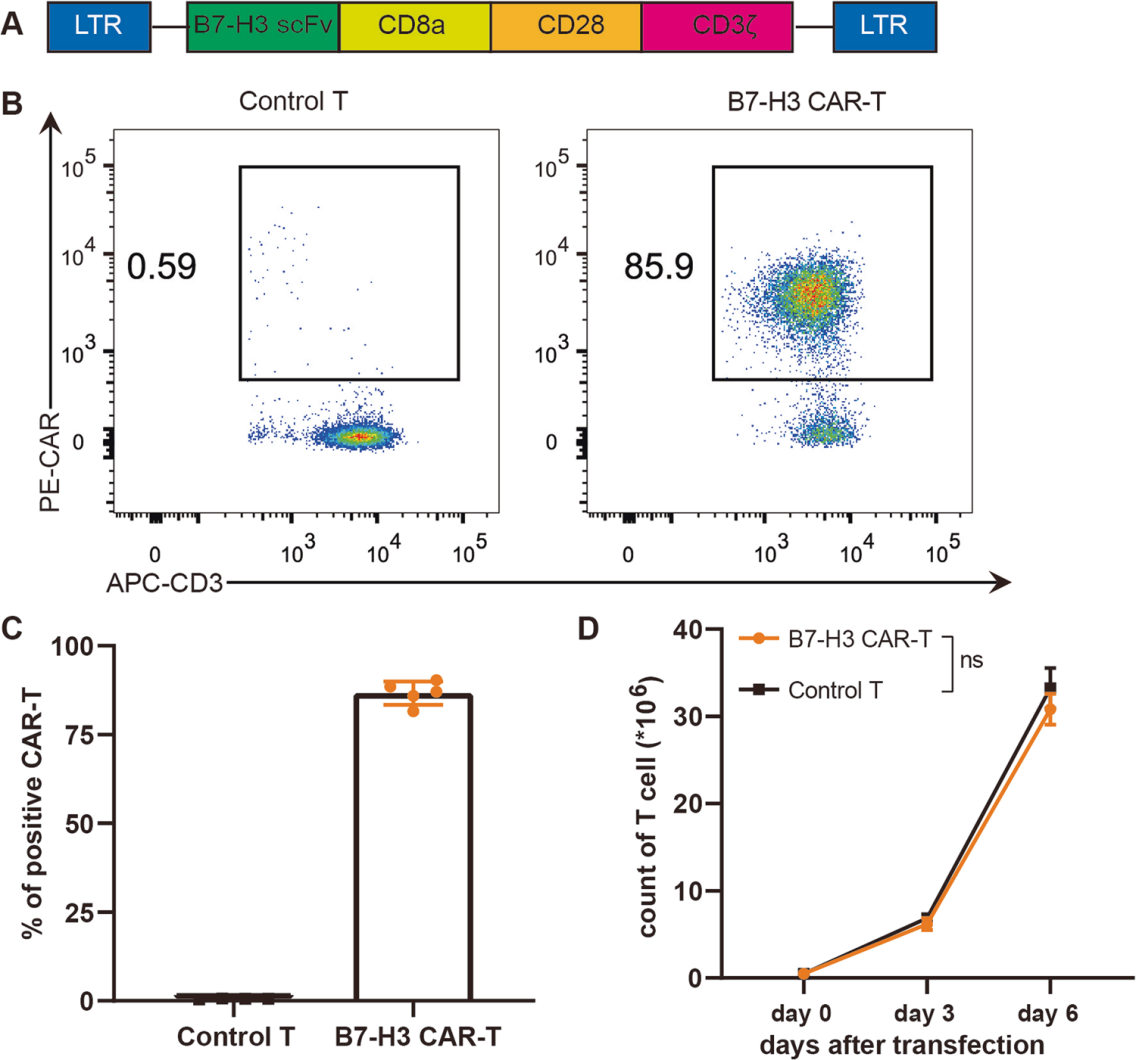
Construction of B7-H3 CAR-T cells. **A** Structural schema of CAR (LTR: long terminal repeat, scFv: single-chain fragment variable). **B** The expression of IgG-Fc on the surface of T cells was detected by flow cytometry to show the transfection efficiency of CAR. **C** Statistical graph of the CAR positive rate of T cells. The data are represented as means ± SEM. **D** Proliferation curves of control T and B7-H3 CAR-T cells. Paired data were analyzed using Student’s *t* test.

We synthesized the B7-H3 scFv sequence based on 8H9 clone and constructed the second-generation B7-H3 CAR containing CD8α transmembrane region, CD28 intracellular costimulatory domain, and CD3ζ intracellular signaling domain after confirming that B7-H3 is highly expressed in prostate cancer (Fig. 2A). B7-H3 CAR-T cells were constructed by infecting CD3/CD28-activated T cells with a retroviral vector encoding B7-H3 CAR, and the activated T cells were used as control T cells. The positive rate of CAR expression on the surface of CAR-T cells reached 85% (Fig. 2B, C). CAR-T cells expanded approximately sixtyfold on the sixth day after transfection. This finding was comparable with that of control T cells, thereby indicating that the proliferation of T cells remained unaffected by the expression of CAR (Fig. 2D).

### B7-H3 redirected CAR-T cells efficiently kill PCa cells

**Fig. 3:**
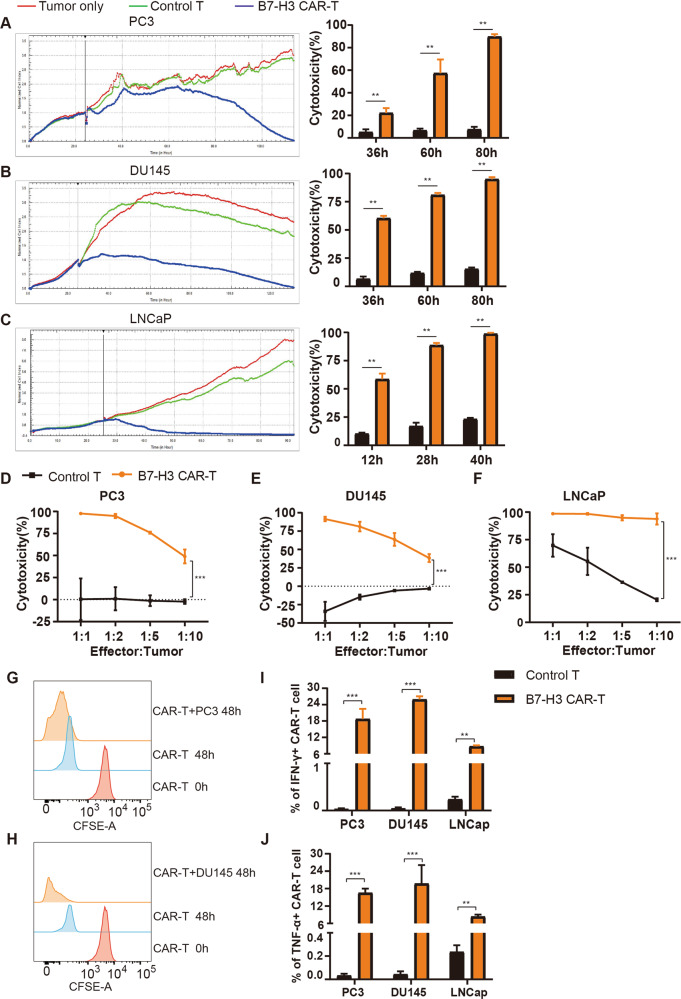
Cytotoxicity of B7-H3 CAR-T cells against prostate cancer cells. **A**–**C**. Cytotoxicity analysis of B7-H3 CAR-T cells against B7-H3 positive target cells by real-time cell assay. The data are represented as the mean cytotoxicity ± SEM. **D**–**F** Flow cytometry detected the final proportion of T cells co-cultured with PC3 (**D**), DU145 (**E**) or LNCaP (**F**), and calculated the percentage of killing. The data are represented as the mean cytotoxicity ± SEM. **G**, **H** Effect of tumor cells on the proliferation of B7-H3 CAR-T cells by FCM; flow cytometry was used to detect the fluorescence intensity of control T and B7-H3 CAR-T cells to reflect the proliferation of T cells after co-incubating CFSE-stained T cells with PC3 (**G**) or DU145 (**H**). **I**, **J** Effect of tumor cells on the cytokine release of B7-H3 CAR-T cells by FCM; T cells were subjected to membrane permeation, fixation, and antibody staining to detect the IFN-γ (**I**) and TNF-α (**J**) levels of control T and B7-H3 CAR-T cells under the stimulation of prostate cancer cells. These data are expressed as the mean percentage of cytokines ± SEM. **P < 0.05, ***P < 0.001.

We tested the function of B7-H3 specific T cells in vitro using FCM and RTCA techniques. Three prostate cancer cells were co-incubated with control T and B7-H3 CAR-T cells at an effector-to-target ratio (E:T) of 1:1. RTCA was used to record the cell index in real time and reflect the adherence and killing of tumor cells. The percentage of surviving CAR-T and tumor cells was analyzed after 4 days of co-culture using flow cytometry to calculate the killing rate. The results of RTCA showed that tumor cells alone as blank group, the effect of control T cells on three B7-H3 positive tumor cells is insignificant. Compared with blank and control T groups, B7-H3 CAR-T cells induced a nearly complete elimination of tumor cells (Fig. 3A–C). The FCM results demonstrated that the B7-H3 CAR-T cell group presents minimal residual tumor cells at a high target ratio, that is, CAR-T cells exert strong killing effects on PC3, DU145, and LNCaP cells. The killing effect was dose-dependent and enhanced with the increase of the effector-to-target ratio (Fig. 3D–F). These results suggested that B7-H3 CAR-T cells exert a significant anti-tumor effect on B7-H3 positive PCa cells.

Control T and CAR-T cells were stained with CFSE and then incubated with PC3 or DU145 cells at an E:T of 1:1. The CFSE fluorescence intensity of T cells was detected via flow cytometry after 48 h, and the fluorescence intensity decreased with the increase of T cell proliferation. As shown in Fig. 3G, H, the fluorescence intensity of control T cells co-cultured with the two tumor cells was the same as that of T cells alone, while the CFSE intensity of B7-H3 CAR-T cells was significantly weaker after co-culture, thereby demonstrating that tumor cells enhance the proliferation of CAR-T cells. These findings showed that B7-H3 CAR-T cells could eradicate tumor cells and tumor cells stimulate CAR-T cells to proliferate efficiently. CAR-T cells activate, proliferate, and release cytokines IFN-γ, TNF-α, Granzyme A, and Granzyme B in the presence of target antigen. Hence, we collected T cells co-cultured with tumor cells at an E:T of 1:5 for 48 h and then detected the release of IFN-γ and TNF-α from CAR-T cells using flow cytometry. The detection of cytokines demonstrated that the PC3, DU145, and LNCaP cells expressing B7-H3 significantly increase the release of IFN-γ (Fig. 3I) and TNF-α (Fig. 3J) from CAR-T cells compared with control T cells.

### B7-H3 CAR-T cells do not kill B7-H3 negative PCa cells

**Fig. 4:**
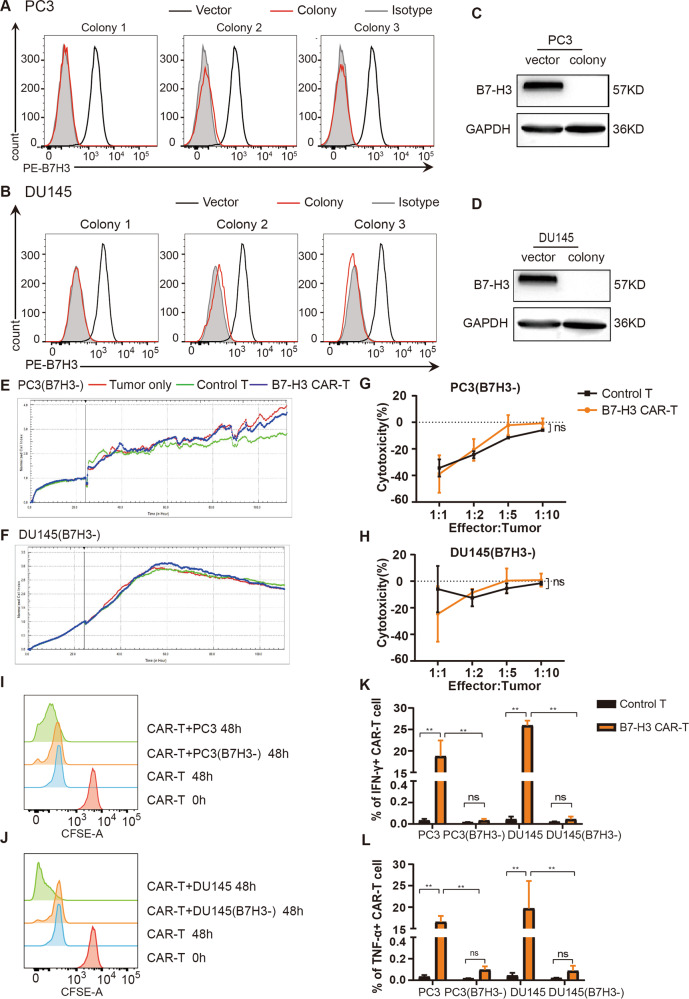
Cytotoxicity of B7-H3 CAR-T cells against B7-H3 negative tumor cells. **A**, **B**. The expression of B7-H3 protein on the surface of PC3(B7H3-) (**A**) and DU145(B7H3-) (**B**) monoclonal cells was detected by flow cytometry. **C**, **D** Western blot was utilized to confirm the expression of B7-H3 in PC3, PC3(B7H3-), DU145, and DU145(B7H3-) cells. **E**, **F** RTCA was used to verify the killing of control T and B7-H3 CAR-T cells to B7-H3 negative PC3(B7H3-) (**E**) and DU145(B7H3-) (**F**), and the right figure is the corresponding statistics. **G**, **H** The killing of PC3(B7H3-) (**G**) or DU145(B7H3-) (**H**) with B7-H3 CAR-T and control T cells was assessed via FCM. The killing effect of T cells on tumor cells was calculated by the mean ± SEM. **I**, **J** CFSE staining was used to detect the proliferation of control T and CAR-T cells co-incubated with PC3(B7H3-) (**I**) or DU145(B7H3-) (**J**). **K**, **L** The release of IFN-γ (**K**) and TNF-α (**L**) from T cells under the stimulation of B7-H3 negative tumor cells. These data are exhibited as the mean percentage of cytokines ± SEM. **P < 0.05.

We used CRISPR-Cas9 technology to knock out B7-H3 in PC3 and DU145 cells and constructed PC3 (B7H3-) and DU145 (B7H3-) cells via puromycin selection and flow sorting to clarify the specificity of B7-H3 CAR-T cells. Flow cytometry was used to detect the expression of B7-H3 on cell clones (Fig. 4A, B), and Western blot was utilized to verify the successful construct of PC3(B7H3-) and DU145(B7H3-) cells (Fig. 4C, D). The effect of B7-H3 CAR-T cells on B7-H3 negative cells was detected by RTCA and flow cytometry. The RTCA curve showed that control T cells do not kill PC3(B7H3-) and DU145(B7H3-) cells. Moreover, the similar curves of B7-H3 CAR-T, blank, and control T groups suggested that B7-H3 directed CAR-T cells present no cytotoxicity against B7-H3 negative tumor cells (Fig. 4E, F). The results of FCM were consistent with those of RTCA (Fig. 4G, H).

As shown in Fig. 4I, J, the CFSE intensity of B7-H3 CAR-T cells co-incubated with PC3(B7H3-) or DU145(B7H3-) cells demonstrated no discernible difference from that of CAR-T cells alone but was higher than the results of co-incubation with PC3/DU145 cells. This finding suggested that the proliferation of B7-H3 CAR-T cells was unaffected by B7-H3 negative PC3 and DU145 cells. Cytokine assays revealed that PC3(B7H3-) and DU145(B7H3-) cells do not promote the release of IFN-γ (Fig. 4K) and TNF-α (Fig. 4L) from B7-H3 CAR-T cells. These results implied that B7-H3 CAR-T cells specifically target and kill B7-H3 positive tumor cells and release cytokines.

### B7-H3 CAR-T cells inhibit the growth of prostate cancer in vivo

**Fig. 5:**
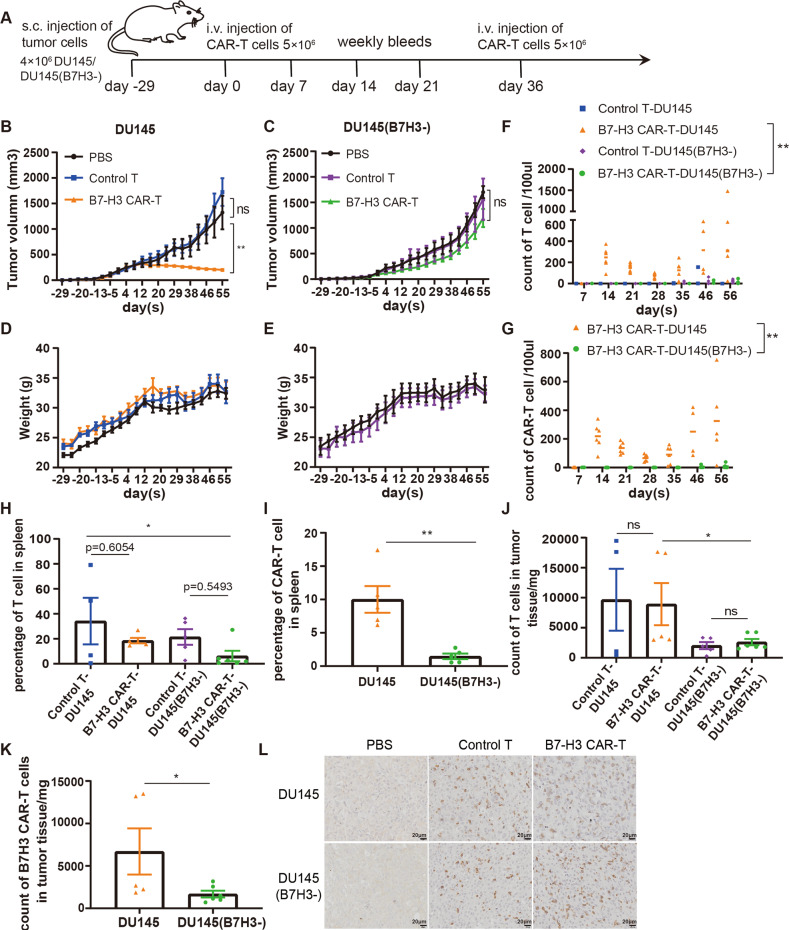
Antitumor effect of B7-H3 CAR-T cells in xenograft mice. **A** Treatment protocol for subcutaneous xenograft tumors, DU145, and DU145 (B7H3-) in NCG mice. **B**, **C** Tumor volume curves of DU145 and DU145(B7H3-) mice treated with PBS (*n* = 4), control T cells (*n* = 5), and B7-H3 CAR-T cells (*n* = 6). The tumor volume data are represented as mean ± SEM (mm^3^). **D**, **E** Body weight curves of the two mouse models. The tumor weight data are represented as mean ± SEM (g). **F**, **G** Content of T cells (Figure F) and CAR-T cells (**G**) in the peripheral blood of mice. **H**, **I** Percentage of T cells (**H**) and CAR-T cells (**I**) in the spleen. **J**, **K** Numbers of T cells (**J**) and CAR-T cells (**K**) in tumor tissues. **L** Immunohistochemical analysis of T cell infiltration in tumor tissue (Scale bar, 20 × 50 μm). All of the results are expressed as the mean of triplicate values ± SEM. **P < 0.01, *P < 0.05 via ANOVA for multiple comparisons of the three treatment groups.

We evaluated the anti-tumor activity of B7-H3 CAR-T cells in vivo given that B7-H3 specific CAR-T cells could effectively eliminate B7-H3 positive PCa cells in vitro. We infused immunodeficient NCG mice with DU145 or DU145(B7H3-) cells to construct subcutaneous xenograft models of prostate cancer (Fig. 5A). The 5 × 10^6^ control T or B7-H3 CAR-T cells were infused via the tail vein on days 29 and 65. Blood was collected from the tail vein every week to detect the content of T and CAR-T cells in peripheral blood after treatment to evaluate the survival of T cells in mice. As shown in Fig. 5B, tumors in the PBS and control T groups of DU145 mice continued to grow while B7-H3 CAR-T cells significantly inhibited the tumor growth (*p* < 0.05). Tumors in the B7-H3 CAR-T group of DU145(B7H3-) mice were slightly smaller than those of PBS and control T groups but statistically insignificant (Fig. 5C). The absence of weight loss in mice in the treatment with control T and B7-H3 CAR-T cells in both models (Fig. 5D, E), demonstrated that B7-H3 CAR-T cells exert no evident toxic or side effects on mice.

The content of T cells in the peripheral blood of DU145 mice exhibited fewer T cells in the control group than that in the B7-H3 CAR-T group (*p* < 0.05), while T cells in both groups of DU145(B7H3-) mice were lower. The contents of T and CAR-T cells in the peripheral blood of the CAR-T group of DU145 mice were higher than those of the CAR-T group of DU145(B7H3-) mice (*p* < 0.01) (Fig. 5F, G). The mouse spleen was taken and ground at the end of the experiment to detect the ratio of T (Fig. 5H) and CAR-T (Fig. 5I) cells. The proportion of spleen T cells between the control T and B7-H3 CAR-T groups of the two mice was the same, and the ratio of T and CAR-T cells in the spleen of DU145(B7H3-) mice was clearly lower than that of DU145 mice. The tumors of mice were ground to measure the content of T and CAR-T cells, and excised tumor tissues were subjected to CD3 immunohistochemical staining to evaluate the infiltration of T cells. The number of T cells in the tumors of the control T and B7-H3 CAR-T groups in the two mouse models was comparable, and the number of T and CAR-T cells in the tumors of B7-H3 knockout mice was less than that of the DU145 mice (*p* < 0.05) (Fig. 5J, K). T cell infiltration in the tumor tissues of the control T and B7-H3 CAR-T groups was evident (Fig. 5L).

Overall, these results verified that B7-H3 specific CAR-T cells inhibit the tumor growth of B7-H3 positive DU145 mice and effectively expand and infiltrate in mice, without obvious side effects.

## Discussion

In this study, we constructed a CAR targeting B7-H3 based on 8H9 clone. We used CRISPR-Cas9 technology to knock out B7-H3 in PC3 and DU145 cells and then verified that B7-H3 CAR-T cells specifically recognize B7-H3 positive target cells and produce cytokines IFN-γ and TNF-α to induce tumor cell lysis in vitro. In vivo, DU145 tumor-bearing mice showed better response to the B7-H3 specific CAR-T cell treatment without obvious side effects.

High heterogeneity of antigens limits the efficacy of CAR-T cells in solid tumor [34]. B7-H3 exhibits limited expression in normal tissues and is widely expressed in tumor cells and vasculature. The overexpression of B7-H3 in some malignancies, such as prostate cancer [35], clear cell renal cancer, lung cancer, and ovarian cancer, is associated with few tumor-infiltrating lymphocytes, low cancer severity, and poor prognosis. B7-H3 is an attractive target for cancer immunotherapy because of its difference between healthy and malignant tissues. The 8H9 antibody has been used to treat a variety of tumors to ensure the efficacy and safety of the treatment when applied to CAR-T cells targeting B7-H3 [36, 37]. Researchers have shown that the B7-H3 antibody-drug conjugate (ADC) eradicates established tumors and metastases, improves overall survival, and has been used in a broad range of anticancer treatments with a favorable safety profile in phase I clinical trials.

This study demonstrated that B7-H3 CAR-T cell therapy is a promising treatment strategy for PCa although some issues still require further investigation. On the one hand, clinical trials are lacking, and the clinical and toxic side effects in humans are still uncertain. Therefore, the safety and efficacy of B7-H3 CAR-T cells in PCa patients need to be explored in the future. On the other hand, the potential of B7-H3 as an anti-metastatic marker for prostate cancer must be examined because it plays a role in cancer migration, invasion, and angiogenesis.

In this study, we validated the antitumor effect of B7-H3 redirected CAR-T cells against PCa in vitro and in vivo. Collectively, our findings provide a new target for the treatment of prostate cancer, and B7-H3 CAR-T cell is a potential treatment for PCa patients.

## Materials and methods

### Clinical samples and cell lines

Clinical samples of prostate tissues were obtained from patients diagnosed with prostate cancer in the Affiliated Hospital of Xuzhou Medical University (XYFY2021-KL325-01). Three PCa cell lines (PC3, DU145, and LNCaP) and 293 T were purchased from the Cell Bank of the Chinese Academy of Sciences. All cell lines used in the experiments were identified by STR and proved to be free of mycoplasma contamination. All cell culture medium contained 10% FBS (fetal bovine serum) and 1% penicillin and streptomycin. Peripheral blood mononuclear cells (PBMCs) were isolated from whole blood via density-gradient centrifugation using Ficoll reagent. Recombinant human IL-7 (rhIL-7, 1‰) and rhIL-15 were added to the medium of T cells. Cells were cultured in a sterile incubator at 37 °C with 5% CO_2_. The study was approved by the Ethics Committee of the Affiliated Hospital of Xuzhou Medical University, and informed consent was obtained from all patients (XYFY2016-JS033-01).

### Reagents

The activator of human T, anti-CD3 monoclonal antibody (OKT3) and anti-CD28 monoclonal antibody (CD28.2), was purchased from Life Technologies. Recombinant human IL-7 and IL-15 were purchased from PrimeGene BioTech, China. RetroNectin was purchased from Takara Bio Inc. (T100A). The following antibodies used for flow cytometry (FCM) were sourced from BioLegend, PE anti-human B7-H3 (331606), APC anti-human CD3 (317318), PE-Cy7 anti-human TNF-α (502930), FITC anti-human IFN-γ (502506) and PE anti-human IgG Fc (410708). Fetal bovine serum, medium, and penicillin/streptomycin were acquired from Gibco. The 5(6)-carboxyfluorescein diacetate succinimidyl ester (CFSE) was purchased from Thermo Fisher Scientific (C34554). Brefeldin A (BFA) was purchased from Biogems. Cytofix/Cytoperm kit was purchased from BD Pharmingen. B7-H3 (14058), GAPDH antibody (649202), and peroxidase-coupled goat anti-rabbit IgG secondary antibody (SA00001-2) for Western blot were bought from CST, BioLegend and Proteintech, respectively.

### Flow cytometry

The expression of B7-H3 on the surface of prostate cancer cells was detected with anti-B7H3 antibody. For the analysis of CAR expression, control T cells or CAR-T cells were incubated with the B7-H3 protein (SinoBiological, 11188-H02H) and then stained with IgG-Fc, followed by anti-CD3 antibody. The proliferation of T cells was evaluated by CFSE staining. B7-H3 CAR-T cells were co-incubated with tumor cells at an effector-to-target (E:T) ratio of 1:5 for 24 h to measure cytokines of CAR-T cells. T cells were fixed, permeabilized and stained with IFN-γ and TNF-α antibodies to measure the intracellular cytokines. B7-H3 gene knockout tumor cell lines were screened by puromycin and sorted with BD FACSAria™ III Cell Sorter. All samples were collected using BD FACSCanto™ II flow cytometer, and the data were processed with FlowJo software.

### Generation of B7-H3 specific CAR-T cells

The scFv sequence based on 8H9 antibody was synthesized by the company, and then introduced into a plasmid vector containing CD8α transmembrane region, CD28, and CD3ζ intracellular signal domains to construct B7-H3 CAR. The B7-H3 CAR and two packaging plasmids were co-transfected into 293T cells. The virus supernatants were collected at 48 and 72 h after transfection, centrifuged at 2000 rpm for 10 min to remove cell debris, and stored at −80 °C for later use. PBMC cells from whole blood were isolated using Ficoll reagent and cultured in L500 medium supplemented with 10% serum, 1% penicillin/streptomycin, and 10 ng/ml rhIL-7 and 5 ng/ml rhIL-15. PBMC cells were activated with 1 μg/ml of CD3/CD28 antibodies-coated 24-well plate for 48 h. The 24-well low-adsorption plate coated with RetroNectin was used to infect T cells with the virus solution and then replaced with L500 growth medium after centrifugation at 30 °C and 1500*g* for 2 h. T cells were expanded and cultured according to a density of (0.7–1) × 10^6^ cells/ml, and the percentage of CAR-positive T cells were detected 48-h later by flow cytometry. Activated T cells without infection served as control cells.

### Cytotoxicity assay

Cytotoxicity of CAR-T cells was assessed with Real-Time Cell Analysis (RTCA) instrument or flow cytometry. In the first step, L500 complete medium (50 μl) was added to each well of the E-plate to detect the background baseline value. Tumor cells (1.5 × 10^4^) were added to each well, and then plate was placed on the instrument for the continuously monitoring of adherence and proliferation of tumor cells through impedance values in the second step. The second step was terminated at an appropriate time. Effector cells were subsequently added to the target cells at a certain effect-to-target ratio to ensure that the activity of CAR-T cells can be directly reflected by cell index. Flow cytometry was applied to detect the ratio of T cells to tumor cells at the beginning and end of co-culture as well as calculate the killing rate of CAR-T cells. Wells with only tumor cells served as the control.

### Cell proliferation assay

Effector cells were stained with 1.5 μM CFSE dye for 10 min at room temperature in the dark and then stopped with an equal volume of serum at 37 °C, and finally washed with PBS containing 2% serum. Flow cytometry was utilized to check whether the staining was successful. Target cells and effector cells were co-cultured at an E:T of 1:5, and a control well without target cells was set at the same time. T cells were collected and the CFSE fluorescence intensity was measured using flow cytometry to evaluate the proliferation of T cells after 48 h. CFSE fluorescence intensity decreased with T cell proliferation. Data were analyzed using FlowJo software.

### Cytokine production assay

Tumor cells and effector cells (control T or CAR-T) were seeded into a 24-well plate at an E:T of 1:5. After co-culture for 24 h, BFA was added to block the extracellular release of cytokines for another 6 h. Then T cells were harvested for CAR staining, fixation, and membrane permeabilization, IFN-γ and TNF-α antibody staining, and detection via flow cytometry.

### CRISPR-mediated B7-H3 gene knockout

The gRNA targeting human B7-H3 (5ʹ-CACCGACCCAGTGGTGGCACTGGT-3ʹ) was derived from the published article [38] and synthesized and introduced into Lenti-CRISPR v2 plasmid. And then lentiviral particles containing B7H3 gRNA were produced in 293T cells. Finally, the viral supernatant was collected to infect PC3 and DU145 tumor cells. After 48 h of infection, monoclonal cells were sorted into 96-well plates, and then cells were expanded and tested for knockout efficacy of B7-H3. Three cell clones were mixed after western blot validation to obtain B7H3 deficient cell lines of PC3 and DU145.

### Western blot

Prostate cancer cells were lysed with lysing buffer containing protease inhibitors to extract total proteins. The protein extracts were separated by 10% SDS-PAGE, transferred to a PVDF membrane, and incubated with antibodies against B7-H3 and GAPDH. Horseradish peroxidase-conjugated goat anti-rabbit IgG was used as the secondary antibody, and the protein content was displayed with enhanced chemiluminescence liquid (ECL).

### Xenograft mouse model

All animal treatment procedures were approved and performed in strict accordance with the regulations of the animal care and use committee of Xuzhou Medical University. Four weeks old immunodeficient NCG male mice were purchased from GemPharmatech Co Ltd and housed in a specific pathogen-free (SPF) animal facility of Xuzhou Medical University. 4 × 10^6^ of prostate DU145 or DU145(B7H3-) cells were resuspended into 150 µl of PBS and subcutaneously inoculated into the lower right limb of mice to construct the prostate xenograft tumor model. The body weight and tumor size of mice were measured every 4 days, and tumor volume was calculated using the formula length × width^2^ × 0.5. Mice were randomly divided into three groups when the tumor volume reached 100–150 mm^3^, and PBS, 5 × 10^6^ B7-H3 CAR-T cells, or equal number of control T cells were infused via the tail vein on days 29 and 65 after tumor injection to assess the effect of B7-H3 CAR-T cells. Blood was collected weekly through the tail vein to detect the proliferation of T cells in peripheral blood during the treatment. Mice were sacrificed at the end of the experiment, and the spleen and tumor tissues were ground to detect the content of T cells and CAR-T cells. Meanwhile, the infiltration of T cells in tumor tissue was assessed by immunohistochemistry. The animal study was approved by the Ethics Committee of Xuzhou Medical University (L20210226419).

### Statistical analysis

All experiments were repeated at least three times, and experimental data were statistically analyzed with Graphad Prism 7.0 software. The data were expressed as mean ± standard error (SEM). Unpaired Student’s *t* test was used to compare two independent samples, and ANOVA was used to compare the significant differences among multiple groups. *P* < 0.05 was considered statistically significant.

## Supplementary information

Original Data File

## Data Availability

All data involved in this study are available in the main text, and we will provide the original data to support the findings upon reasonable request. Materials can be made available through agreement with the corresponding author.
