# Supplementary figures and images for: B7-H3 specific CAR-T cells exhibit potent activity against prostate cancer
Source: Cell Death Discov. 2023 May 6;9:147. doi: 10.1038/s41420-023-01453-7 (PMC10164129; doi:10.1038/s41420-023-01453-7)

**Figure 4C、4D**

B7-H3


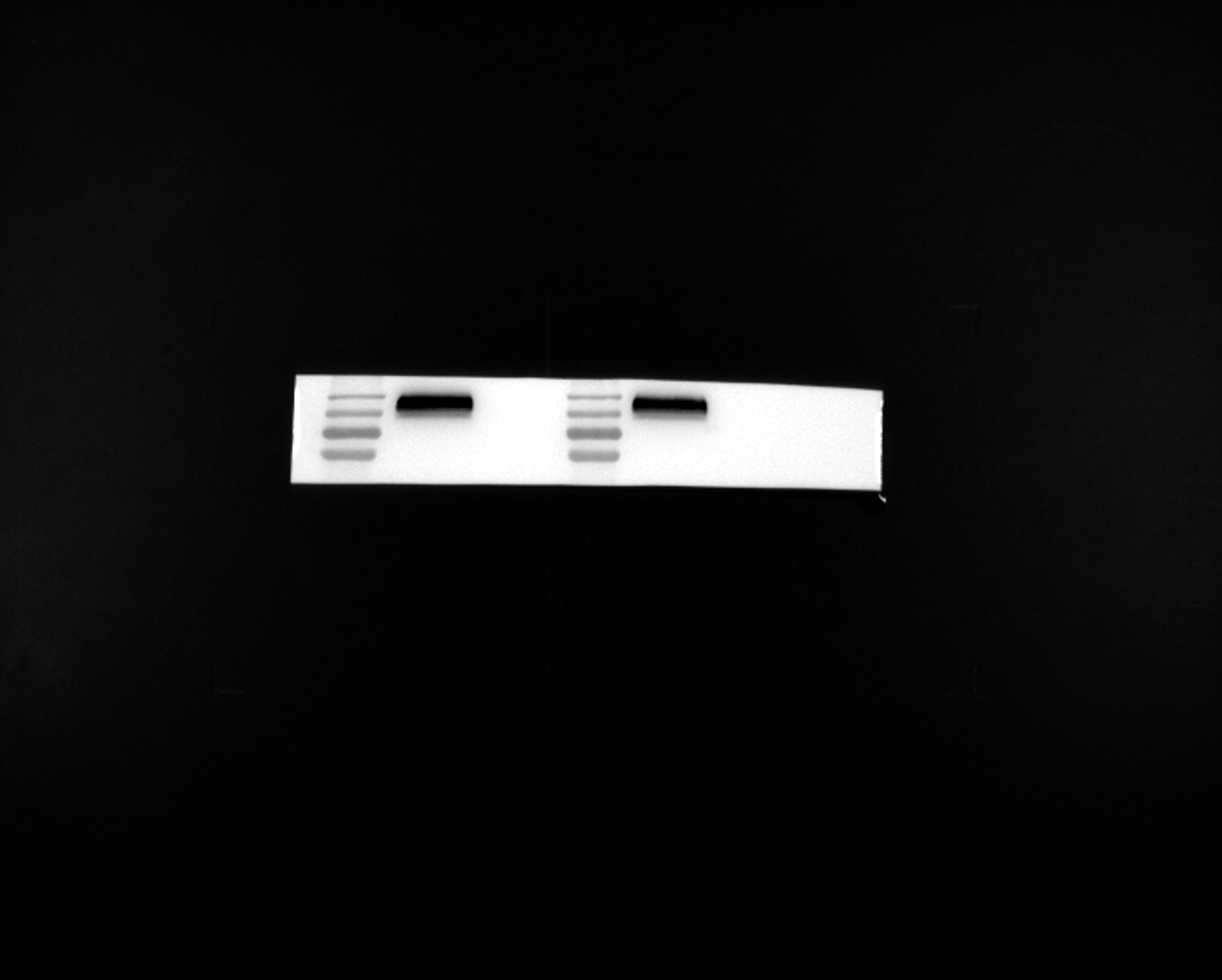


GAPDH


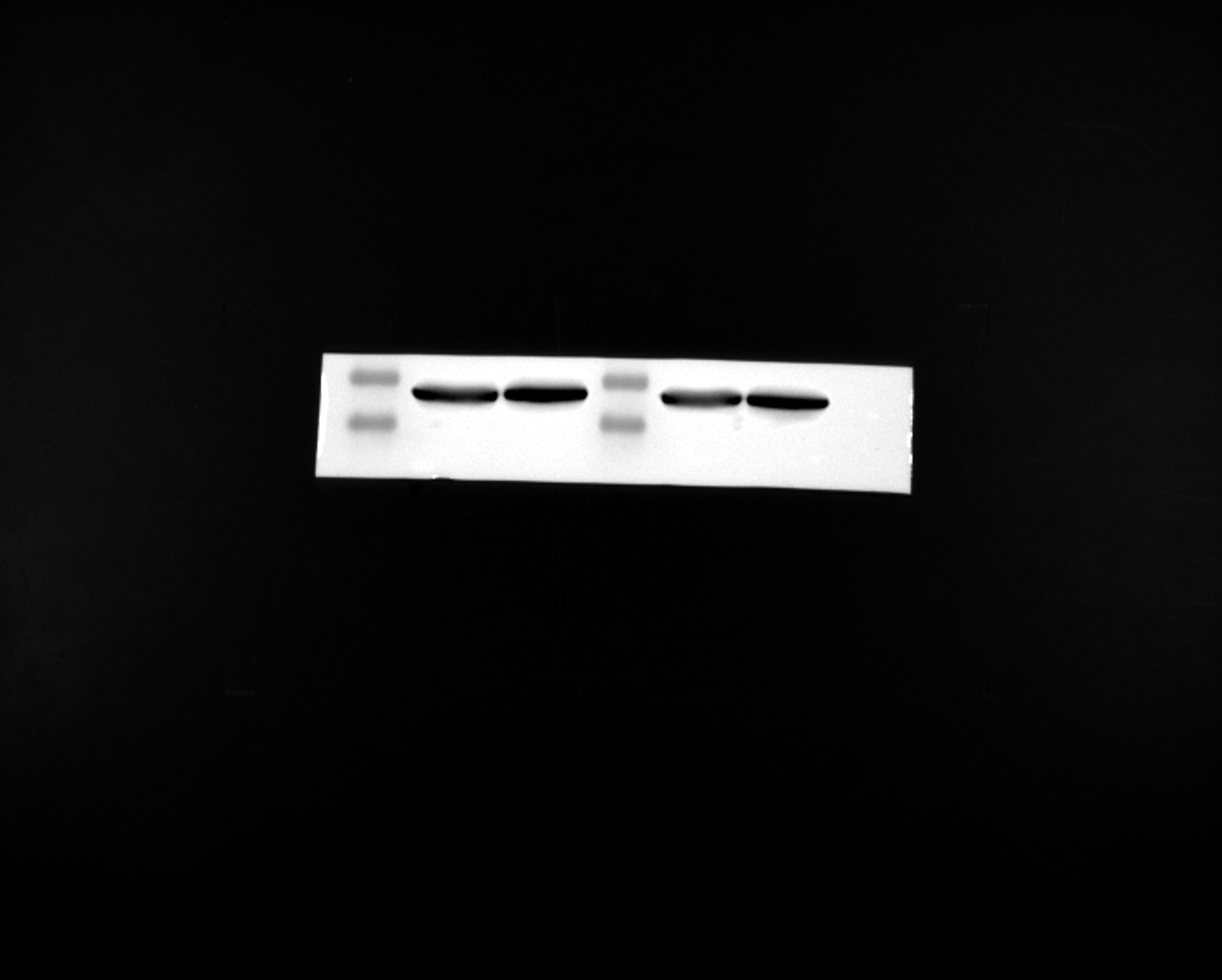


The original images for figure 4C、4D.

Left to right: PC3, PC3(B7H3-), DU145, DU145(B7H3-)

Supplement: Supplementary file 1 — Original Data File [file 41420_2023_1453_MOESM1_ESM.docx]
